# Genetic Improvement in Sunflower Breeding—Integrated Omics Approach

**DOI:** 10.3390/plants10061150

**Published:** 2021-06-04

**Authors:** Milan Jocković, Siniša Jocić, Sandra Cvejić, Ana Marjanović-Jeromela, Jelena Jocković, Aleksandra Radanović, Dragana Miladinović

**Affiliations:** 1 Institute of Field and Vegetable Crops, Maksima Gorkog 30, 21000 Novi Sad, Serbia; sinisa.jocic@ifvcns.ns.ac.rs (S.J.); sandra.cvejic@ifvcns.ns.ac.rs (S.C.); ana.jeromela@ifvcns.ns.ac.rs (A.M.-J.); aleksandra.dimitrijevic@ifvcns.ns.ac.rs (A.R.); dragana.miladinovic@ifvcns.ns.ac.rs (D.M.); 2 Department of Biology and Ecology, Faculty of Sciences, University of Novi Sad, Dositeja Obradovića 3, 21000 Novi Sad, Serbia; jelena.lazarevic@dbe.uns.ac.rs

**Keywords:** sunflower, genomics, epigenomics, transcriptomics, proteomics, integrated omics

## Abstract

Foresight in climate change and the challenges ahead requires a systematic approach to sunflower breeding that will encompass all available technologies. There is a great scarcity of desirable genetic variation, which is in fact undiscovered because it has not been sufficiently researched as detection and designing favorable genetic variation largely depends on thorough genome sequencing through broad and deep resequencing. Basic exploration of genomes is insufficient to find insight about important physiological and molecular mechanisms unique to crops. That is why integrating information from genomics, epigenomics, transcriptomics, proteomics, metabolomics and phenomics enables a comprehensive understanding of the molecular mechanisms in the background of architecture of many important quantitative traits. Omics technologies offer novel possibilities for deciphering the complex pathways and molecular profiling through the level of systems biology and can provide important answers that can be utilized for more efficient breeding of sunflower. In this review, we present omics profiling approaches in order to address their possibilities and usefulness as a potential breeding tools in sunflower genetic improvement.

## 1. Introduction

Crop production today is threatened by severe abiotic stresses due to extreme weather conditions (droughts, floods and other disasters), accompanied by emerging diseases and a decrease in arable land [1,2,3]. Certainly, the most important mission in agriculture is to provide sufficient quantities of plant based products for a growing world population. The benefits in yield and food quality brought by “The Green Revolution” are far from enough to keep up with the pace as the increasingly growing demand forecasts an increase of 70% for food requirements by 2050 [4]. Projection of linear progress of 2% of genetic gain in order to meet demands is questionable as, so far, annual gain in crop productivity is rated from 0.8 to 1.2%, which is considered insufficient [5].

Giving importance to the production of healthy food for human consumption, sunflower (*Helianthus annuus* L.) has been recognized as a major source of high-quality edible oil and dietary fibers [6,7]. Native to North America, with its exceptional ability for adaptability to different climatic and soil conditions, sunflower is grown around the globe as a crop that significantly contributes in vegetable oil consumption. In addition to its basic application in human nutrition, sunflower oil has a wide range of applications as a supplement in chemical and pharmaceutical industries [8]. The main sunflower breeding goals are aimed towards high seed and oil yield, genetic resistance and high level of tolerance to the economically most important diseases, insects and parasitic weed (broomrape), as well as tolerance to abiotic stresses (in the first place to drought). As one of the most important oilseed crops worldwide, in order to meet growing global demands for sunflower products, intensified efforts for implementation of all available advanced breeding tools are required to improve the quantity and the quality of sunflower output by focusing on factors that are limiting phenotype expression of genetic potential. Special attention should be paid to the complexity in inheritance of the afore-mentioned traits, especially for resistance and tolerance to different pests and drought. Thus far, substantial progress has been made in order to improve breeding process in sunflower by application of DNA markers, especially for disease resistance [9] and tolerance to abiotic stress [10].

Further progress in sunflower improvement relies on combining all available cutting-edge scientific tools, techniques and platforms in modern breeding such as molecular breeding [11], genomics [12,13,14] and other functional omics [15,16,17,18,19] through integrated approach with the aim to thoroughly decipher the complex mechanisms in the background of many important agronomic traits. Integrated omics approach through the systems biology offers novel possibilities for deciphering the complex pathways, and molecular profiling provides important answers that can be utilized for more efficient breeding of sunflower. That is why we here address achievements and knowledge in increasingly applicable omics approaches as a potential breeding tool for sunflower genetic improvement.

## 2. Molecular Omics Profiling

### 2.1. Genomics—Pangenomics

The genetic improvement of any crop, and thus of sunflower, largely depends on the acquisition of appropriate variability as the main cornerstone that allows successful progress as a response to new breeding challenges. Additionally, detailed information on genetic and phenotypic data of available germplasm is important from the breeding aspect in terms of correct selection of material for crossing. In this regard, one of the drawbacks is the insufficient information on existing genetic resources, which has the consequence that although there is a very large collection of genetic material around the world, there is a lack in discovering beneficial alleles that can be utilized in breeding and transferred into elite genotypes [3,20]. As previously outlined, detection of favorable genetic variation largely depends on thorough genome sequencing through broad and deep resequencing and construction of pangenome, in order to characterize in detail diverse germplasm, thus providing clear profile view of the locked genetic variation [11]. Evolution of sequence technologies, so called Next-Generation Sequencing technologies (NGS), enabled construction of high-quality, chromosome-level assembly of several plant genomes [3]. These NGS technologies evolved from short reads with limited capacity to map structural variants to long-read sequencing techniques, which enabled exploring the huge genetic diversity present across diverse accessions [21,22,23]. Since the development of the first genetic map on wild sunflower in 1993, the evolution of molecular markers enabled the successive addition of new markers to the map and enabled the positioning and detection of desirable genes on individual linking groups [24,25,26,27]. Finally, a high-quality reference for the sunflower genome is available (Table 1) that contains 3.6 gigabases consisting of long and highly similar repeats and allows more efficient exploitation of sunflower genetic background towards improvement in biotic and abiotic stress resistance, as well as oil production [28].

**Figure 1:**
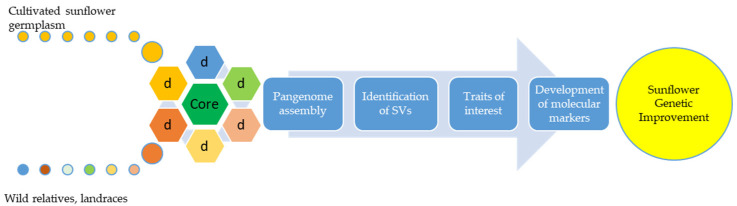
The basic concept of pangenome applied to sunflower. The concept of pangenome can be used for broadening genetic diversity in the pursuit for important traits. Using biotechnological tools, molecular markers can be developed for the appropriate trait and used in breeding to improve sunflower genetics.

As indicated in the previous study, an assembly of three high-quality sunflower reference genomes is available, two of them covering genomes of inbred lines XRQ and HA412-HO and one of the restorer line PSC8 [29]. These high resolution sunflower maps enable narrowing the targeted area in the pursuit for desirable genes for many important traits [30]. In order to obtain more complete information on the total genetic variability of a particular species, the concept of pangenome has recently been disguised (Figure 1). It is based on the fact that the total genetic variation of a population or species consists of a core genome that represents a set of genes that are common to all individuals and a dispensable genome consisting of a small number of genes that are absent in one or more individuals [31,32].

Cultivated sunflower is related to a large number of wild sunflower species. Wild relatives are an invaluable source of desirable genes, especially for resistance to biotic and abiotic stresses, which is lost during the domestication process of sunflower. By applying technological progress, it is possible to overcome difficulties in the use of wild species [33]. As a useful example, CRISPR–Cas9 genome editing strategy was applied for editing several loci important for yield and productivity in cultivated tomato lines and enabled de novo domestication of wild tomato [34]. Later, the pan-genome of wild relatives of cultivated soybean was established by sequencing and de novo assembly of seven phylogenetically and geographically representative accessions [35]. These authors revealed evidence of variation of agronomic traits such as biotic resistance, seed composition, flowering and maturity time, organ size and final biomass. The great usefulness of this concept is reflected not only in a better understanding of the domestication process, but also in the knowledge that many agronomically important traits are controlled by larger structural variations [36]. Pangenome approach has been assembled for various important cultivated plant species [37,38,39,40]. In order to quantify genetic contributions from wild relatives in cultivated sunflower pangenome, a study was conducted in order to sequence and analyze 493 accessions of diverse origin [41]. The authors succeeded in assembling a pangenome for cultivated sunflower comprised of 61,205 genes (Table 1). By comparison of assembled genes with wild relatives, they were able to identify introgressed genomic regions from wild sunflower species. This study provided valuable insight that mostly genes from introgressed regions are found to be associated with resistance to biotic stress (downy mildew). Pangenomic studies usually include a limited set of accessions. Given the goal to cover as much genetic variation within a genus as possible, it is proposed to include a maximum of diverse accessions of each species in a pangenomic study. By generating genetic background with different origin, it is possible to provide a more comprehensive picture of genetic diversity.

### 2.2. Epigenomics

During their life cycle, plants are exposed to different types of challenges, among which various environmental stresses have a severe impact on phenotype development. This primarily includes stresses such as extreme temperatures and lack of moisture and thus nutrient availability, and what makes them an extremely difficult opponent is their unpredictability. Due to the significant influence of external factors, plants have developed various mechanisms that help them cope with constant challenges to a certain extent. These mechanisms, of course, represent genetic and epigenetic modifications, helping them to survive the challenges they are exposed to [42]. Knowledge about these complex mechanisms requires thorough studies about epigenetic changes, which involve DNA methylation, histone modifications, chromatin remodeling and activity of small RNAs (sRNAs) as they represent insufficiently known variables [43,44]. These reversible modifications of the genomic DNA have significant functions in gene management and cell activities [45]. Epigenetic studies have gained wings in recent years through high-throughput assays and provided evidence of the role of epigenetic DNA marks on phenotypic expression of several traits [3]. Although several epigenetic marks (known as tags) have been discovered, the mainly characterized ones are DNA methylation and histone modification [45]. Studies on model plant (Arabidopsis) found that DNA methylation affects important processes such as seed development and gametogenesis, as well as flowering time and root length [46,47,48]. Moreover, plant ageing and senescence is found to be under the influence of alterations in chromatin structure as evidenced by the fact that the age of plant tissue affects the variation in the level of DNA methylation [49]. Excluding roles in developmental processes, DNA methylation and histone modification are also involved in plant responses to environmental challenges as well as in immunity response to turnip mosaic virus [50,51,52]. Diseases are one of the main limiting factors in sunflower production, and therefore finding epigenetic markers is of great benefit in breeding for resistance. Since sunflower is usually exposed to a combined attack of several pathogens, preliminary study on finding epigenetic marks for resistance to the combined attack of downy mildew and broomrape has been established [53]. The study showed the possible role of defensin in the immune response of sunflower to a combined attack of these constraints. High concentrations of heavy metals are one of the common phenomena that affect the growth and development of plants, and sunflower as well. An earlier study outlined that zinc (Zn) concentrations are found to influence DNA methylation patterns in sunflower seedlings growth and development [54]. Considering that the utilization of heterosis in sunflower hybrids, and in hybrids in general, depends on the genetic distance between the parent pairs, DNA methylation level can be used as an indicator of hybrid vigor in sunflower [55]. Results from the study indicated epigenetic divergence between sterile A-lines, restorer lines and their diallel hybrids with different values (57%, 46% and 50%, respectively) for full methylation state. Additionally, differences in methylation pattern pointed to greater heterogeneity between parental lines than between their hybrids. Use of epigenetic marks requires their stable inheritance across generations, and thus information regarding stability and heritability of those marks is important in order to be applicable in breeding process [56]. An earlier, comprehensive study outlined that epigenetic information can be successfully transmitted to offspring via cytosine DNA methylation [57]. Furthermore, epigenetic variations in DNA methylation are inducing epiallelic diversity, which is responsible for phenotypic variation via changes in transcription and morphology. The possibility to screen and transfer these modifications provides an opportunity to increase genetic divergence and thus phenotypic variations, which can be implemented for agronomic improvement of important crops. An impressive example of successful implementation of epigenetic modifications is documented in a previous study [58] where authors tested the potential of MutS HOMOLOG1 (MSH1) system for generating valuable epigenetic variation in soybean. Derived MSH1 populations showed an increase in variance for important agronomic traits as well as higher yield stability. The results from the aforementioned studies provide opportunities for new approaches for enhancement of sunflower breeding programs as the information about stability and heritability of epigenetic tags is of immense importance in order to be applicable in crop improvement.

## 3. Gene Function Translation

### 3.1. Transcriptomics

Crops are exposed to an increase in environmental pressure, thus complicating the already sufficiently challenging breeding process. Knowing the biological processes at the molecular level in the life cycle of the plant and within the cell helps us to understand the influence of various factors on plant development. Advanced sequencing technologies allowed high throughput transcriptomic analysis and decoding complex transcriptional changes in phenotype (genotype x environment) development. Data obtained from transcriptome analysis allow identification of transcriptional regulatory elements as well as mechanisms of transcriptional regulation, which are essential in cellular life. The great importance of transcriptomic analysis is reflected in the fact that it provides clearer knowledge about the molecular mechanisms of interaction and genetic basis of plant resistance to disease with the possibility to implement the obtained data in breeding processes for development of resistant genotypes [59]. Transcriptomic study in plants is usually performed for analyzing diverse effects of stresses in order to explain dynamic and complex processes on molecular level, which lead to modifications in plant tissues [60,61,62,63]. Identifying complex transcriptional changes in tissues is possible through identification of transcription regulatory elements and deciphering the mechanisms on which transcription regulation is based [64,65]. Considering that plants are often exposed to various external pressures, comparative transcriptome analyses for plants exposed to different stresses is useful for understanding the role of commonly regulated genes under combined and individual stresses and utilization of such genes in breeding process for combined stress tolerance [66,67]. Meta-analysis was successfully applied for evaluation of contrasting oxidative stress tolerance in sunflower and identified a number of oxidative stress responsive genes that are shared across stresses [66]. The significance of transcriptomic study is also reflected in the fact that it can be used for comparative analysis of genome differences between cultivated crops and wild relatives, thus revealing specific genes that can be important for improvement in the breeding process. Using a long read technique (Iso-seq) as well as RNA sequencing (RNA-seq), transcriptomic basis for salt tolerance and disease resistance of wild sunflower species (*Helianthus argophyllus*) was studied [68]. Authors assembled a high quality reference transcriptome for *H. argophyllus* with over 50,000 genes and found that 205 of them are not present in cultivated sunflower. Transcriptomic response to salt stress revealed more than double the number of genes (3930) that were significantly regulated in root compared to leaf (1885), which is understandable considering the higher sensitivity of root, as it is more exposed to this type of stress [68]. Transcriptomic analysis can be very useful for distinguishing gene expression in drought-sensitive and drought-tolerant genotypes [69], which is of great help for the development of drought-tolerant genotypes as one of the main goals in sunflower breeding. There is an excellent example of successful implementation of transcriptomic data in genomic prediction of complex traits in maize where authors emphasized that transcriptomic data outperformed genetic markers and identified more genes for important agronomic trait and outlined usefulness for predicting complex traits whose variation cannot be accurately identified at the sequence level [70]. More recently, a method based on pooled single-molecule transcriptomics for the discovery of self-incompatibility genes in wild sunflower has been developed, thus providing a useful approach for simultaneous identification of balanced polymorphisms [71].

### 3.2. Proteomics

One of the main targets in sunflower breeding is development of hybrids tolerant to abiotic stresses, with the drought stress being one of the most important [72]. Thus far, conventional breeding methods have been successfully used for development of sunflower genotypes adapted to different eco-environments and to different types of abiotic and biotic stress [73]. Breeding for tolerance to abiotic stresses is especially difficult because it is affected by multiple, interacting mechanisms which have not been thoroughly studied, so there is a lack of knowledge of the complex nature of stress itself. Proteomic analysis is a powerful approach for more comprehensive knowledge about gene expression and their functional mechanisms during plant life cycle. This approach is usefully applied in order to study the functions of proteins in biochemical processes caused by plant reaction to abiotic and biotic stresses [74,75,76]. Such investigations were employed in sunflower in order to elucidate the proteomic basis of cold acclimatization phenomenon, tolerance to drought and contamination with different concentrations of cadmium (Cd), nickel (Ni) and zinc (Zn) [77,78,79]. Comparative proteomic study in sunflower was applied in order to elucidate the complex mechanism of resistance to *Orobanche cumana* [80]. The study identified more than 3500 proteins and provided valuable insights into pathways regarding resistant and susceptible interaction with the possibility to establish more durable resistance against this extremely important obligate parasite. Advances in technology shifted proteomics into a cutting edge powerful tool able to provide insight into mechanisms involved in plant responses to stress on molecular level [81]. These large scale studies are capable to provide quantification of the entire complement of proteins, unravel cellular location, post-transcriptional and post-translational modifications (PTMs), as well as protein–protein interactions [76,82,83]. Likewise, the proteomic approach offers novel possibilities for identification of genes responsible for phenotype response to different challenges during growth and development as besides structural role, proteins are involved in regulation of plant epigenome, transcriptome, and metabolome [83]. Identification of novel genes can be accomplished using marker assisted selection (MAS) and enhances plant breeding programs. Such an approach was already employed to identify protein quantity loci (PQL) [84]. The phenomenon of heterosis is mostly used in sunflower production and in this regard, gel-free proteomic has been applied in order to investigate heterosis profile of sunflower leaves [85]. The study revealed that the effect of heterosis increases input energy via enhanced carbon fixation. More recently, proteomic study on 144 sunflower plants evaluated molecular basis of heterosis effect as well as adaptation to drought [10]. The authors analyzed leaf proteome of 24 sunflower genotypes cultivated in different water regimes using outdoor phenotyping platform Heliaphen and were able to identify more than 3000 proteins and quantify more than one third of them (1211). Proteomics on sunflower is not a sufficiently researched area, but the possibilities provided by this scientific technique, with identification of novel genes, can enable further progress in genetic enhancement of sunflower, especially regarding tolerance to stress factors.

### 3.3. Metabolomics

Described as a missing link in functional genomics, metabolomics is used for precise analysis of complex pool of cellular metabolites, thus providing detailed information about cell functionality. A huge number of metabolites are known in the plant metabolome pool, which exceeds 200,000 [86]. The main field of research in metabolomics is focused towards the identification and quantification of these small molecules involved in plant growth and development, interactions within an organism and responses to environment [87,88]. Metabolomics in plants uses high throughput analysis for a wide range of metabolite mixtures in order to do separation, characterization and quantification of plant extract compounds [89]. Quantitative plant metabolomic analysis is usually driven by nuclear magnetic resonance (NMR) and mass spectrometry (MS) with Fourier transform infrared spectroscopy (FTIR) gaining popularity lately due to technological improvement [89,90,91]. Information from metabolomics is useful for understanding the complex metabolic network, providing deeper insight into the fundamental nature of plant phenotypes in relation to gene role in the metabolic pathways [92,93]. Earlier metabolome study provided comprehensive details about contrasting behavior of sunflower genotypes to sclerotinia (head form) infection and identified 63 metabolites in sunflower head, as the main entry point of pathogen [94]. In a well-documented study, metabolic activity of uniseriate linear glandular trichomes in sunflower was investigated [95]. Using spectroscopic analysis, the authors successfully detected sesquiterpenes and flavonoids, some of which were not previously described and reported. More recent application of metabolomics is involved in discovering metabolic markers that can be used for crop improvement. This idea was brought by earlier study which emphasized high predictive power of metabolites as a biomarker for biomass accumulation and enhancement in plant breeding [96], and their usefulness in crop improvement was also confirmed by more recent studies [90,97,98]. The possibilities offered by these markers in sunflower selection were also investigated [19]. Authors combined targeted and non-targeted metabolomics on sunflower leaves with the aim to find the smallest set of biomarkers which can be used for differentiation of sunflower hybrid components (line, restorer and maintainer) and to distinguish lines grown in well-watered conditions and subjected to stress (drought). The application of LC-MS technique in metabolite detection was documented in a comprehensive study which analyzed the effect of abiotic stress on metabolite groups in sunflower [99]. Results indicated that exposure of sunflower plants to hexavalent chromium (Cr (VI)) caused increase in synthesis of secondary metabolites among which sesquiterpenes and isocoumarins are set aside for more detailed identification in future studies.

### 3.4. Phenomics

Phenotype variations represent a complex network of interactions between genotypes and a multitude of external factors. In order to better understand the genetic basis of a complex trait, a detailed and precise assessment of the phenotype is necessary. Phenotypes with desirable characteristics represent sources of important alleles, which can be identified using high throughput sequencing [100]. Much effort has been put into advancing sequencing technologies to enrich genotypic information, while phenotyping has progressed more slowly, leading to limited characterization of complex quantitative traits [101,102]. Presented as “the next challenge” in the first decade of the 21st century, phenomics is a study for high-throughput and high-dimensional analysis of phenotypic variation [100,103]. Advances in technology have enabled phenomics to explore multivariate phenotypic information, and the use of high-performance computing enables simultaneous processing of a number of phenotypic parameters [102,104,105]. The main purpose of such a system is efficient and precise phenotyping in order to obtain as much data as possible on genes associated with important agronomic traits for more efficient breeding. A large number of phenomic platforms are available that focus on different characteristics, depending on whether phenotyping is done in controlled or field conditions, as well as whether phenotyping is done at the level of the whole plant or at the level of tissues and cells, which is a lot more demanding [106,107]. Breeding for herbicide tolerance is one of the main goals in sunflower breeding, and proper assessment of plant damage manifested by leaf chlorosis is very important for the selection of tolerant genotypes. In this purpose, in order to quantify imidazolinone-induced chlorosis on leaves of sunflower plantlets, Tomato Analyzer color test software has been applied [108]. Sunflower broomrape (*Orobanche cumana*) is one of the main constraints that threatens sunflower production. With constant emergence of more virulent races, breeding for resistant genotypes is of the utmost importance. Although the presence of broomrape stalks becomes evident with the appearance above the ground, development of a fast method for underground screening can accelerate selection of resistant genotypes and improve breeding success. In this direction, progress has been made with the use of blue-green fluorescence (BGF) and thermal imaging as non-destructive monitoring methods for underground detection of the infection by broomrape [109]. Bearing in mind that the tolerance of genotypes to stressful conditions largely depends on the condition of the root system, phenotyping platforms for non-invasive root analysis can be very helpful in breeding for modified root characteristics. Preliminary study for sunflower root phenotyping implemented automated phenotyping platform GROWSCREEN-Rhizo and quantified root traits with the image processing software GrowScreen-Root [110]. Considering that sunflower has a large number of applications among which is also as an ornamental plant, Flower Color Image Analysis (FloCIA) software for sunflower ray floret digital image segmentation and automatic classification has been developed [111]. Recently, an automated device for determining the shape and color of sunflower seeds has also been developed [112].

## 4. Integrated Omics Approach—Systems Biology

**Figure 2:**
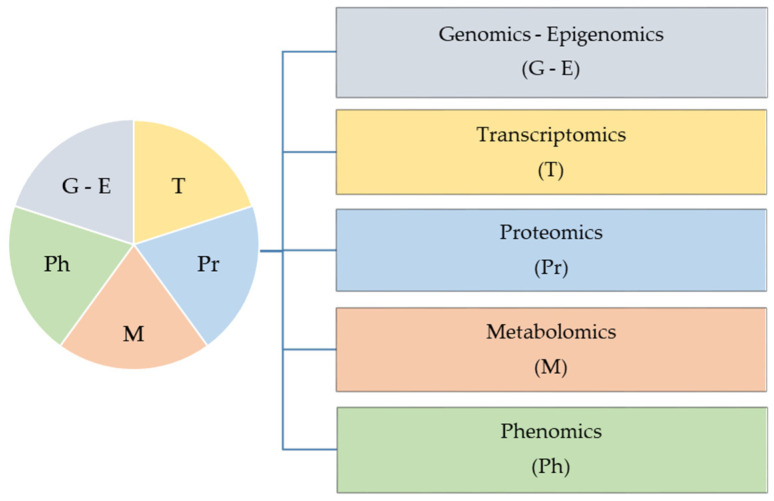
Concept of integrated omics approach, combining benefits of each omic technology. The application of genomics and epigenomics enables the identification of genetic diversity, transcriptomics is used to decode complex transcriptional changes, proteomics allows identification and quantification of post-transcriptional and post-translational modifications, metabolomics is applied for the identification and quantification of metabolites, whereas phenomics is capable for precise physiological biochemical and morphological characterization of genotypes.

Technological developments enabled detailed and complex omic-studies and provided valuable inferences generated by examining the functioning of the cellular system under different circumstances. Such data can be utilized to formulate predictive models of behavior of important agronomic traits and integrate them within the concept of quantitative genetics [3]. As outlined, omics data are expected to group interaction information within and between different biological layers and enhance the predictive ability of a particular trait [113]. An integrative approach, which includes data from different omics datasets, is known as systems biology [114,115]. The basic concept of integrated approach is presented on Figure 2.

What is unique in the systems biology is that the object of observation is viewed as a comprehensive whole of all processes that occur at the molecular level as a result of genetics and interaction with various factors, as well as consequences that occur as a result of interactions. In this way, it is possible to elucidate the complex mechanisms of biological systems and give insight into expression levels of transcripts, proteins and metabolites [116]. One of the flaws of this system is a confusion with the correct interpretation of a huge amount of omics data, often without a clear connection [117]. To overcome incorrect interpretation of data, systematic multi-omics integration (MOI) with a well-defined scheme for linking different data is proposed. The concept of MOI is based on three levels that differ in complexity [117]. Complexity increases moving to a higher level, with level 3 being the most complex, as it is based on mathematical integration with differential and genome scale analyses. As reviewed in a previous study, the integration of omics techniques is compulsory for dissection and thorough knowledge about senescence phenomenon of organs such as flower, fruit and roots [45]. In this regard, a combined transcriptomic and metabolomic study in an integrated approach was applied with the aim to thoroughly characterize the process of leaf senescence in sunflower and provide clearer insight about molecular mechanisms and candidate genes involved in the process [118]. The same approach has been applied in sunflower in order to identify pathways related to hub transcription factors involved in drought stress response [119]. Using a systems biology approach, in the aforementioned study, researchers were able to identify pathways and hubs transcription factors regulated during drought conditions in sunflower. Certainly, the most revolutionary aspect of the application of omics data is the possibility of application in breeding prediction, especially for complex quantitative traits such as seed yield. Hybrid breeding in sunflower is based on crossing CMS and Rf parental lines and testing their combining abilities. It is laborious work because every year a large number of parental lines are crossed in order to make hybrid combinations, which are then tested for seed and oil yield. Proper selection of parent pairs for crossing is certainly the most challenging part in creating new hybrids, and reducing the number of crosses and increasing success is what we strive for by applying information from different data. An excellent example of the application of the integrated approach is given in a comprehensive study where different predictors from genomic, transcriptomic and metabolomic data, measured on maize parent lines, were applied in order to predict the effects of untested hybrids for important quantitative traits [113]. As an outcome, combining omics with genomic data improved predictive ability, while in comparison between predictors, transcriptomic data outperformed others. This is explained by the expression quantitative trait loci (eQTL) analysis, as it showed that transcriptomic data represents more complementary genetic information relative to the expressed gene.

## 5. Conclusions and Prospects

Advances in molecular research and the availability of vast amounts of genomic data offer opportunities to overcome breeding challenges and ensure the advancement of sunflower, as an important source of edible oil. In this regard, a detailed characterization of genotypic and phenotypic diversity is very important in order to have a clearer insight into the variations of important traits, which can be utilized to improve sunflower genetics. The application of omics technologies, such as genomics, epigenomics, transcriptomics, proteomics, metabolomics and phenomics, through an integrated approach, known as systems biology, offers new possibilities to decipher complex pathways and molecular profiling for important and complex agronomic traits and provides answers that can facilitate and improve sunflower. In order to overcome the difficulties that may arise from the application of a large number of obtained data, it is necessary to define an appropriate scheme for analyzing, interpreting and linking the data. Additionally, the use of high-performance computational analysis offers opportunities to enable a deeper comprehension of the gene regulatory network for a given trait and design a more efficient breeding strategy [120]. Lately, intensive research to improve prediction accuracy has resulted in the extensive use of different machine learning techniques. Being based on how humans learn and process information, machine learning is a powerful tool for processing complex data for accurate prediction and have already been used for precision breeding [121]. Although the results of the reviewed omic techniques seem very exciting, they require very expensive equipment that is only available to a small number of scientific institutions. Considering the future advancement of sunflower as one of the most important oil crops worldwide, integrated model implementation should also include sunflower researcher community from different institutions and development of effective teamwork with the aim of doing a comprehensive integration of available technologies and data information as the best way to provide beneficial prospect.

## Figures and Tables

**Table 1:** Sunflower genome and pangenome main characteristics.

Composition Type	Accessions	Strategy	Size	Reference
*Genome*	Inbred line XRQ	102× sequencing coverage of the genome of the inbred line XRQ using 407 single-molecule real-time (SMRT) cells on the PacBio RS II platform.	52,232 protein-coding genes 5803 spliced long non-coding RNAs	[28]
*Pangenome*	493 sunflower accessions which include: 287 cultivated lines, 17 Native American landraces and 189 wild accessions representing 11 compatibile wild species	Pangenome assembled through de novo assembly of unmapped reads	61,205 genes	[41]

## Data Availability

Not applicable.
